# Clusterin exacerbates interleukin-1β-induced inflammation via suppressing PI3K/Akt pathway in human fibroblast-like synoviocytes of knee osteoarthritis

**DOI:** 10.1038/s41598-022-14295-7

**Published:** 2022-06-15

**Authors:** Tachatra Ungsudechachai, Sittisak Honsawek, Jiraphun Jittikoon, Wanvisa Udomsinprasert

**Affiliations:** 1 Department of Biochemistry, Faculty of Pharmacy, Mahidol University, 447 Sri-Ayudthaya Road, Rajathevi, Bangkok, 10400 Thailand; 2 Department of Biochemistry, Osteoarthritis and Musculoskeleton Research Unit, Faculty of Medicine, Chulalongkorn University, King Chulalongkorn Memorial Hospital, Thai Red Cross Society, Bangkok, Thailand

**Keywords:** Biomarkers, Molecular medicine

## Abstract

This study aimed to examine, a multifaceted chaperon-like protein exerting anti-inflammatory action, clusterin (CLU), mRNA and protein levels in the systemic and local joint environment of knee osteoarthritis (OA) patients and to determine whether CLU inhibited interleukin (IL)-1β-induced inflammation in knee OA fibroblast-like synoviocytes (FLSs) through modulating phosphatidylinositol-3-kinase (PI3K)/Akt signaling pathway. CLU protein and mRNA expressions in the synovium and its protein levels in plasma and synovial fluid of knee OA patients were measured using immunohistochemistry, real-time PCR, and ELISA, respectively. Anti-inflammatory effect of CLU was further elucidated in knee OA FLSs treated with IL-1β in the absence or presence of CLU, CLU alone, or PI3K inhibitor (LY294002) along with IL-1β and CLU. In a clinical study, compared with knee OA patients without synovitis, CLU protein and mRNA were expressed in the synovium of knee OA patients with synovitis, especially those with high-grade, consistent with analyses of its plasma and synovial fluid levels. CLU mRNA and protein levels were both associated with synovitis severity. An in vitro study uncovered that CLU significantly alleviated IL-1β-induced overproduction of nitric oxide and IL-6 in knee OA FLSs. Furthermore, CLU significantly attenuated inflammation and extracellular matrix degradation induced by IL-1β via down-regulating expressions of *IL-6*, nuclear factor kappa B, and matrix metalloproteinase-13. Mechanistically, CLU significantly impeded IL-1β-induced Akt phosphorylation in knee OA FLSs, in line with addition of LY294002 along with IL-1β and CLU. These findings suggest that CLU may have potential as a novel therapeutic target for synovitis and cartilage destruction in knee OA.

## Introduction

Knee osteoarthritis (OA) is a degenerative joint disease with high prevalence among the elderly and a major cause of severe pain and disability towards total knee replacement (TKR), thereby representing an enormous healthcare and socioeconomic burden. The disease is pathologically characterized by cartilage degradation, subchondral bone sclerosis, osteophyte formation, and synovial inflammation (synovitis)^1,2^. Although current therapeutic strategies for knee OA focusing on pain relief all simply target the symptoms of disease^3–6^, novel therapies are needed to inhibit the processes driving knee OA pathology possibly due to poorly defined mechanisms underlying knee OA. Given that synovitis is increasingly recognized as a common pathological feature observed in both early and late knee OA, unravelling the mechanism underlying synovitis holds promise for development of new disease-modifying therapies^7,8^. During an inflammatory process, fibroblast-like synoviocytes (FLSs) resided in the inflamed synovium can produce a large number of inflammatory cytokines, chemokines, and matrix-degrading molecules implicated in cartilage degradation^7,9^. Mechanistically, phosphatidylinositol-3-kinase (PI3K)/Akt activation has been reportedly implicated in development of synovitis, degeneration of cartilage, and susceptibility to knee OA^10,11^. It is noteworthy that the molecules known to participate in inflammatory process mediated through PI3K/Akt signaling would be of great interest for exploring their potential as therapeutic targets for knee OA.

Of various molecules responsible for inflammatory response, clusterin (CLU), a multifunctional chaperon-like protein at the crossroad of inflammation and autoimmunity^12–14^, is becoming increasingly recognized as a possible mediator for synovitis in knee OA. As to its anti-inflammatory action, CLU can negatively regulate nuclear factor-kappa B (NF-κB) signaling pathway through stabilization of IκBs^15^. As an upstream molecule of NF-κB pathway, PI3K/Akt plays a regulatory role in NF-κB activation involved in stimulating transcriptional activity of immune and inflammatory processes in response to both injury and infection, thereby plausibly establishing an anti-inflammatory effect of CLU mediated through PI3K/Akt/NF-κB signaling pathway. A more recent study emphasized its significant involvement in inflammatory arthritides, in which CLU overexpression was detectable in the synovium and synovial fluid of patients with either OA or rheumatoid arthritis (RA), and CLU knockdown with small interfering RNA reportedly enhanced production of inflammatory mediators including interleukin (IL)-1β and IL-6 in RA FLSs^16^. These previous findings lend support to the view that CLU may protect against inflammation in synovial tissues and FLSs of knee OA via inhibiting PI3K/Akt signaling pathway.

Whilst the literature suggests anti-inflammatory effect of CLU on various cells, to the best of our knowledge, there are no existing studies to capture the breadth of its precise role in knee OA FLSs. In order to address its beneficial action as an anti-inflammatory molecule in knee OA synovitis, we aimed to determine whether CLU has a protective effect against IL-1β-induced inflammation in knee OA FLSs through counteracting activation of PI3K/Akt signaling pathway and to investigate its mRNA and protein levels in the systemic and local joint environment of knee OA patients with and without synovitis.

## Methods

### Patients and synovial biopsies

The study protocol conducted in conformity with the guidelines of the Declaration of Helsinki was approved by the Institutional Review Board of the Faculty of Medicine, Chulalongkorn University (IRB number 533/54) and the Faculty of Dentistry/Faculty of Pharmacy, Mahidol University (MU-DT/PY-IRB 2019/074.2511). All participants were fully informed regarding the study protocol and procedures prior to entering the study. Written informed consent was obtained from the participants.

Synovial biopsies were harvested surgically at the time of an arthroplasty or TKR from 50 knee OA patients. All knee OA subjects were diagnosed with knee OA in accordance with the criteria of the American College of Rheumatology (ACR). None had underlying diseases such as diabetes mellitus, advanced liver or renal diseases, histories of long-term steroid treatment, other forms of arthritis, previous knee injury and/or infection, malignancy, or other chronic inflammatory diseases.

### Hematoxylin and eosin (H&E) staining

**Figure 1:**
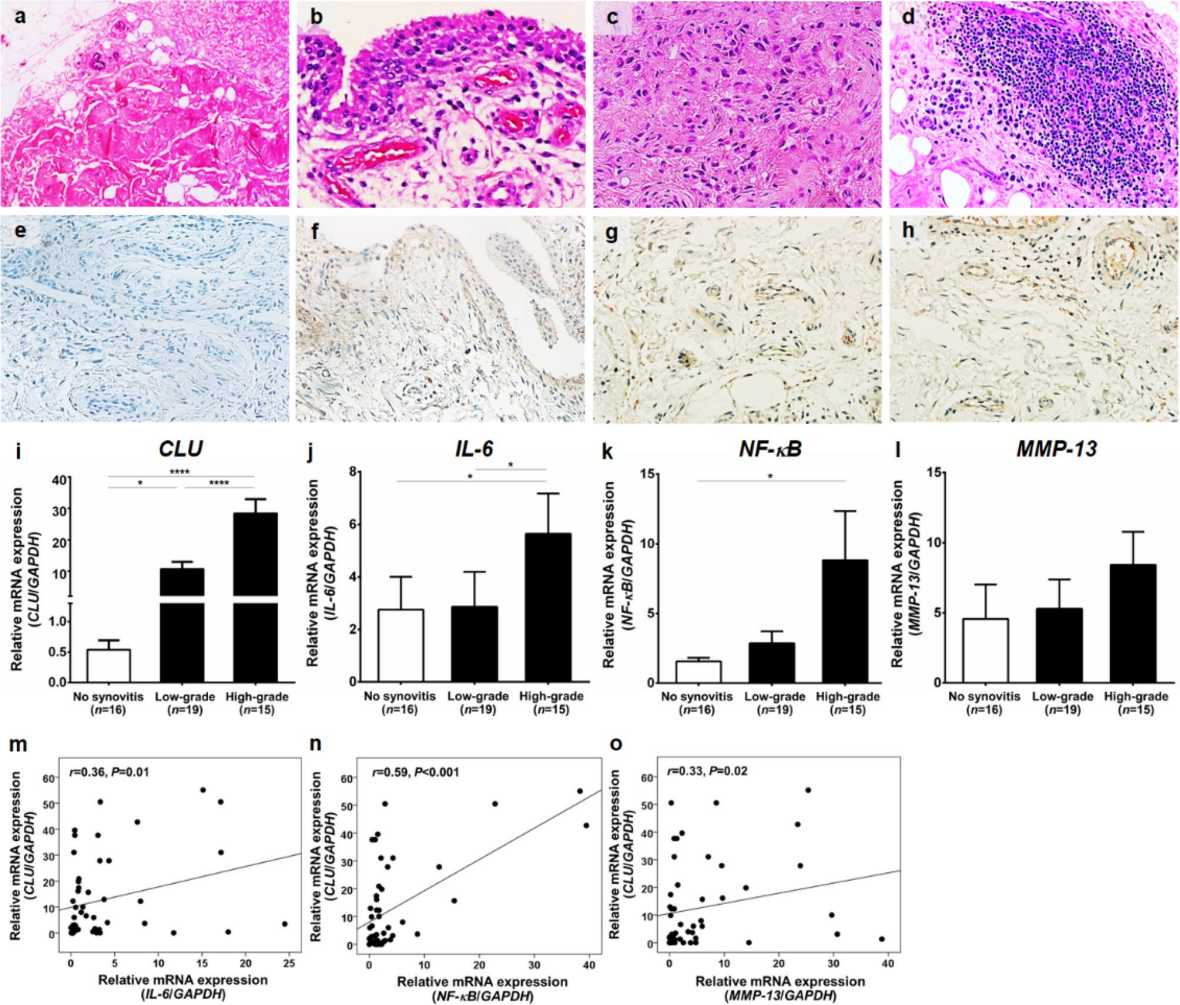
Histopathological staining and *CLU* mRNA in the synovium of knee OA patients. (**a**) Knee OA synovium without synovitis. (**b**) Knee OA synovium with synovitis showing hyperplasia of synovial lining layer. (**c**) Increased density of stromal cell. (**d**) Inflammatory infiltrate. (**e**) Immunohistological staining displaying scarce expression of CLU protein in knee OA synovium without synovitis. (**f**) CLU protein overexpression in the synovial lining layer of knee OA synovium with synovitis. (**g**) CLU protein overexpression in the sublining layer of knee OA synovium with synovitis, especially in stromal cell. (**h**) CLU protein overexpression in the sublining layer of knee OA synovium with synovitis, especially in inflammatory cells. (**i**) Relative *CLU* mRNA expression in knee OA synovium. (**j**) Relative *IL-6* mRNA expression in knee OA synovium. (**k**) Relative *NF-κB* mRNA expression in knee OA synovium. (**l**) Relative *MMP-13* mRNA expression in knee OA synovium. (**m**) Positive association of *CLU* with *IL-6*. (**n**) With *NF-κB*. (**o**) With *MMP-13*. *P < 0.05, ****P < 0.0001 for comparisons among knee OA with different groups with regard to synovitis severity.

Synovial tissues from 50 knee OA patients were paraffin-embedded and subsequently sectioned, according to standard protocols. The tissue sections were routinely stained with H&E to determine morphological changes in the synovium. To semi-quantify degree of synovitis in knee OA synovium, 3 histopathological features of each OA synovium were evaluated by a pathologist who was blinded to clinical status and diagnosis of the patients, according to the following relevant morphological alterations: hyperplasia of synovial lining layer, increased density of stromal cell, and inflammatory infiltrate, as depicted in Fig. 1a**–**d. All defined histopathological features are scored from 0 to 3, in which 3 averages were summed to create a total score of synovitis ranging from 0 to 9. The parameters of synovitis scoring system were summarized, as follows: 0–1, no synovitis; 2–4, low-grade synovitis; and 5–9, high-grade synovitis, as previously described^17^.

### Immunohistochemistry

Immunohistochemical staining was conducted to determine localization of CLU protein expression in synovial tissues of knee OA patients. Using an autostainer (Ventana Medical Systems Inc., Tucson, AZ, USA), a standard immunohistochemical technique was performed. In a brief manner, tissue sections were deparaffinized and rehydrated. For 10 min, 0.3% hydrogen peroxide was used to inhibit endogenous peroxidase activity. After 5 min of heat-induced antigen retrieval in 10 mmol/L citrate buffer (pH 6), the slides were treated for 7 min in pepsin and subsequently incubated for 2 h with 1:200 diluted primary antibodies (Abcam, Cambridge, MA, USA). Following that, the sections were stained for 45 min at room temperature with the secondary antibody coupled to streptavidin/horseradish peroxidase. 3,3-Diaminobenzidine tetrahydrochloride (Sigma, St. Louis, MO, USA) was utilized to visualize reaction products.

### Cell isolation and treatment

Primary FLSs were isolated from synovial tissue samples derived from 6 patients with knee OA who underwent TKR using enzymatic digestion. Briefly, the samples were aseptically minced into small pieces, followed by sequential digestion with 0.1% trypsin in phosphate buffer solution (PBS) for 30 min and 3.5 mg/mL collagenase II (Invitrogen, Carlsbad, CA) in Dulbecco’s modified Eagle medium (DMEM, Hyclone Laboratories Inc., South Logan, UT) supplemented with antibiotics and 10% fetal bovine serum (FBS, Hyclone Laboratories Inc., South Logan, UT) at 37 °C, 5% CO_2_, and 95% humidity for 2 h. After collagenase digestion, the cells were then recovered through centrifugation to yield a cellular pellet that was re-suspended and maintained in DMEM containing antibiotics and 10% FBS. The cells were cultured at 37 °C with 5% CO_2_, and the culture medium was changed every 2–3 days. For subsequent experiments, FLSs at the first or second passage (P1-P2) were used and treated, as follows: (1) FLSs without any treatment as a control group, (2) FLSs treated with 10 ng/mL IL-1β (R&D Systems, Greater Minneapolis, MN, USA), (3) FLSs treated with recombinant CLU (R&D Systems, Greater Minneapolis, MN, USA) at different concentrations (1, 5, 10, or 20 μg/mL), (4) FLSs treated with a combination of 10 ng/mL IL-1β and CLU, and (5) FLSs pre-treated with 10 μM PI3K inhibitor (LY294002, R&D Systems, Greater Minneapolis, MN, USA) along with 10 ng/mL IL-1β and CLU.

### 3-(4,-Dimethylthiazol-2-y)-2,5-diphenyl-tetrazolium bromide (MTT) assay

To investigate cell viability following treatment with various concentrations of CLU and to explore whether CLU could subdue IL-1β-induced cytotoxicity in knee OA FLSs, viability of knee OA FLSs was assessed using MTT assay (Sigma-Aldrich, St. Louis, MO, USA). In brief, isolated FLSs were seeded at a concentration of 5.0 × 10^4^ cells/mL into 96-well plates containing serum-free DMEM and then incubated at 37 °C, 5% CO_2_, and 95% humidity for 24 h. After starvation in serum-free DMEM, knee OA FLSs were stimulated with IL-1β, CLU, IL-1β-CLU combination, LY294002 combined with IL-1β and CLU, or without any treatment for 24 h. Subsequently, sterile MTT was added into the wells and incubated for 4 h. Thereafter, the culture medium was removed, and DMSO was then added. Afterwards, the plate was gently shaken for 10 min until the purple crystals were completely dissolved. The absorbance was measured using an automated microplate reader at 450 nm.

### Quantitative real-time polymerase chain reaction (PCR)

Total ribonucleic acid (RNA) was extracted from synovial tissues of knee OA patients and from knee OA FLSs at a concentration of 2.0 × 10^4^ cells/mL in 24-well plates following stimulation for 24 h using RNeasy Mini Kit (Qiagen, Hilden, Germany), with cDNA reverse transcribed using TaqMan Reverse Transcription Reagents (Applied Biosystems, Inc., Foster City, CA, USA). To examine relative mRNA expressions of *CLU*, *IL-6*, *NF-κB*, and matrix metalloproteinase-13 (*MMP-13*) in knee OA synovium and to investigate relative mRNA expressions of inflammatory and catabolic mediators including *IL-6*, *NF-κB*, and *MMP-13* in knee OA FLSs, real-time PCR was performed using QPCR Green Master Mix HRox (Biotechrabbit GmbH, Hennigsdorf, Germany) on StepOnePlus Real-Time PCR System (Applied Biosystems, Inc., Foster City, CA, USA). Primers used for *CLU*, *IL-6*, *NF-κB*, *MMP-13*, and glyceraldehyde 3-phosphate dehydrogenase (*GADPH*) amplification are demonstrated in Supplementary Table 1. Relative mRNA expressions of *CLU*, *IL-6*, *NF-κB*, and *MMP-13* normalized to *GADPH* as an internal control were determined using 2^−∆∆Ct^ method.

### Enzyme-linked immunosorbent assay (ELISA)

Plasma and synovial fluid samples were obtained from knee OA patients during surgery when the arthroscopy or TKR was performed and stored immediately at − 80 °C for later measurement. After treatment for 24 h, the culture medium was collected from knee OA FLSs seeded at a concentration of 2.0 × 10^4^ cells/mL into 24-well plates and subsequently kept at − 80 °C until utilized. Plasma and synovial fluid CLU levels in knee OA patients and IL-6 levels in the culture medium acquired from knee OA FLSs were quantitatively measured using a commercial ELISA kit (R&D Systems, Minneapolis, MN, USA), according to the manufacturer's instructions.

### Phospho-Akt/total Akt ELISA

Phosphorylated and total Akt levels in cell lysates derived from knee OA FLSs at concentration of 2.0 × 10^4^ cells/mL in 24-well plates after treatment for 24 h were measured using a commercial ELISA kit, according to the manufacturer’s instructions (Abcam, Cambridge, MA, USA).

### Total nitric oxide (NO) assay

After stimulation for 24 h, the culture medium from knee OA FLSs at a concentration of 2.0 × 10^4^ cells/mL in 24-well plates was harvested to determine total NO levels indicating reactive oxygen species (ROS) production using the Nitrate/Nitrite Fluorometric Assay Kit (Abnova, San Francisco, CA, USA), according to the manufacturer’s instructions. The fluorescence of each sample was read at 375 nm excitation and 417 nm emission using an automated microplate reader.

### Statistical analysis

All statistical analyses were executed by SPSS Statistics version 26.0 (SPSS Inc., Chicago, IL, US) and GraphPad Prism 8.0 (GraphPad Software, Inc., CA, US). Demographic and clinical characteristics among groups were evaluated using Chi-square tests and one-way analysis of variance (ANOVA) where appropriate. Comparisons in means among each group were evaluated by ANOVA with a Tukey post hoc test, while Kruskal–Wallis *H* test was utilized for comparison of abnormally distributed continuous variables. Correlations between CLU levels and clinical parameters were accomplished with Spearman’s rank correlation coefficient test (*r*). Multivariate logistic regression analysis with adjustments for confounding factors was conducted to determine the independent associations. Data are represented as mean ± standard deviation (SD). A two-tailed *P*-value < 0.05 was considered for statistically significant differences and correlations.

## Results

### Clinical characteristics of study participants

Baseline demographic characteristics of knee OA patients are summarized in Supplementary Table 2. On the basis of histopathological classification for synovitis severity, knee OA patients were classified into 3 groups: the patients with no synovitis (0–1, *n* = 16), low-grade synovitis (2–4, *n* = 19), and high-grade synovitis (5–9, *n* = 15). Mean age, gender ratio, and body mass index (BMI) values among knee OA patients with different groups were not significantly different.

### CLU protein and mRNA expressions were increased in knee OA patients with synovitis

We firstly determined CLU protein expression in knee OA synovium with and without synovitis using immunohistochemistry. As illustrated in Fig. 1a–d, H&E staining was performed to determine histological features of knee OA synovium with and without synovitis. In knee OA synovium with synovitis, histopathological findings showed hypertrophy of the synovial lining layer (Fig. 1a), presence of synovial stoma (Fig. 1b), and infiltration with inflammatory cells (Fig. 1c). In contrary to the inflamed synovium, absences of enlargement of the lining layer, synovial stoma, and infiltration of inflammatory cells were all found in knee OA synovium without synovitis (Fig. 1d). For CLU protein expression, immunohistochemical staining depicted that CLU expression was detected in both the synovial lining layer and the synovial sublining layer of the inflamed synovium (Fig. 1f–h), inconsistent with the analysis in the non-inflamed synovium showing scarce expression of CLU demonstrated as faint cytoplasmic staining (Fig. 1e).

Apart from CLU protein expression analysis, we subsequently examined relative mRNA expressions of *CLU* and signaling molecules involved in NF-κB pathway including *IL-6*, *NF-κB*, and *MMP-13* in knee OA synovium using quantitative real-time PCR. Compared with knee OA patients without synovitis, relative *CLU* mRNA expression was found to be significantly elevated in the synovium of the patients with high- and low-grade synovitis (*P* < 0.0001, *P* < 0.05, respectively) (Fig. 1i). Correspondingly, knee OA patients with high-grade synovitis exhibited significantly higher relative *CLU* mRNA expression in the synovium than those with low-grade synovitis (*P* < 0.0001). In stratified analyses, relative *IL-6* mRNA expression was significantly up-regulated in the synovium of knee OA patients with high-grade synovitis, as compared to those without synovitis (*P* < 0.05) (Fig. 1j), consistent with *NF-κB* mRNA expression analysis (*P* < 0.05) (Fig. 1k). Alternatively, there were no significant differences in relative *MMP-13* mRNA expression among groups (Fig. 1l). Interestingly, further analysis depicted positive relationships between *CLU* mRNA expression and mRNA expressions of *IL-6* (*r* = 0.36, *P* < 0.05), *NF-κB* (*r* = 0.59, *P* < 0.001), and *MMP-13* (*r* = 0.33, *P* < 0.05) in the synovium of knee OA patients (Fig. 1m–o).

**Figure 2:**
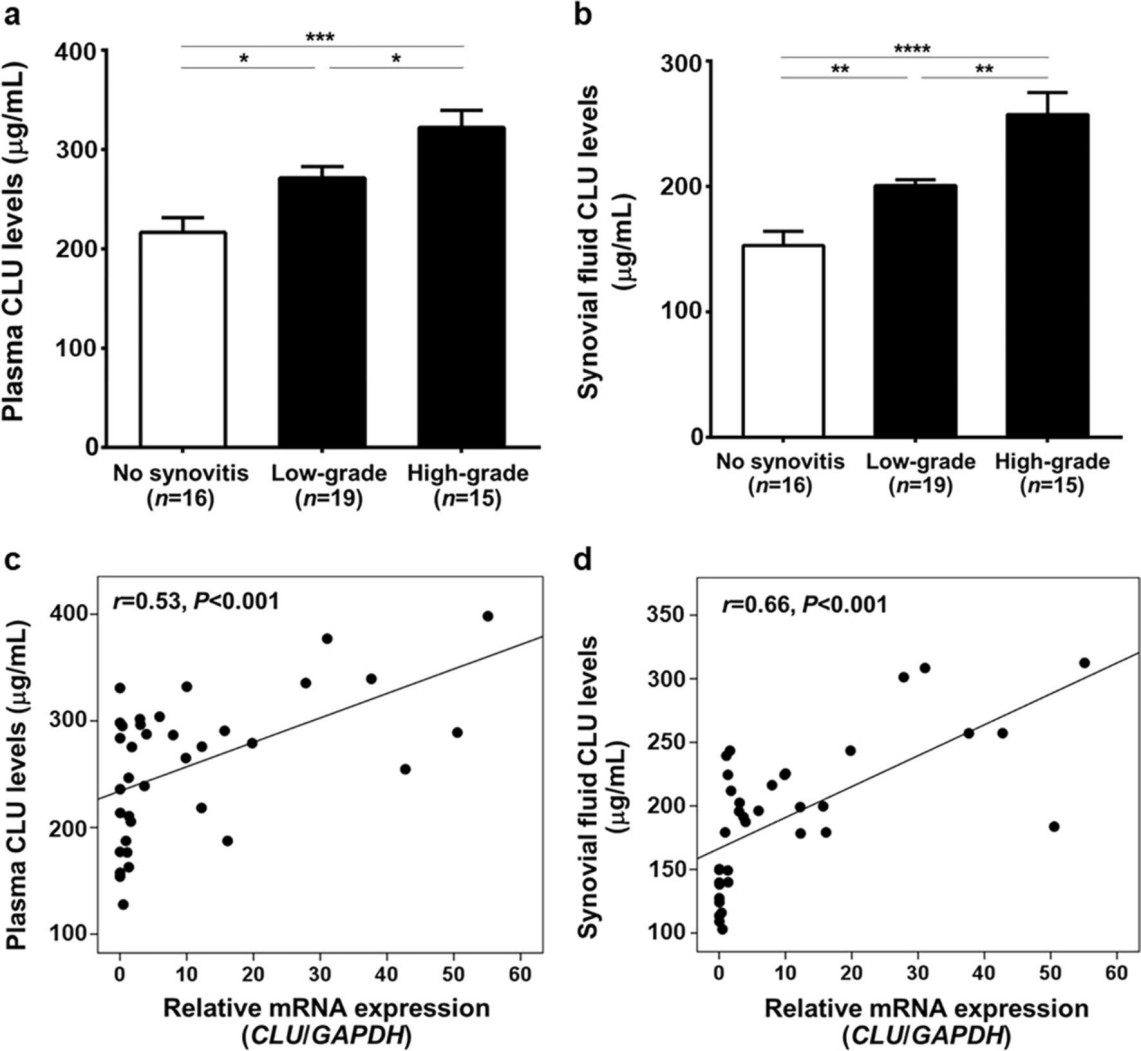
CLU protein levels in the circulation and synovial fluid of knee OA patients with different groups based on degree of synovitis. (**a**) Plasma CLU levels. (**b**) Synovial fluid of CLU levels. (**c**) Direct link between plasma CLU and its mRNA expression. (**d**) Positive association between synovial fluid CLU and its mRNA expression. *P < 0.05, **P < 0.01, ***P < 0.001, ****P < 0.0001 for comparisons among knee OA with different groups with regard to synovitis severity.

In addition to CLU protein and mRNA expressions in knee OA synovitis, its protein levels in the circulation and synovial fluid of knee OA patients with different synovitis groups were further determined. As revealed in Fig. 2a,b, plasma CLU levels in knee OA patients with high-grade synovitis were significantly greater than those in the patients with low-grade synovitis and without synovitis (*P* < 0.05, *P* < 0.001, respectively), which were in line with synovial fluid CLU levels (*P* < 0.01, *P* < 0.0001, respectively). Notably, additional analysis unveiled direct associations of CLU protein levels in plasma and joint fluid of knee OA patients with its mRNA expression in knee OA synovium (*r* = 0.53, *P* < 0.001; *r* = 0.66, *P* < 0.001, respectively) (Fig. 2c,d). Supporting the above-mentioned findings, our previous study showed significant increases in circulating and synovial fluid CLU levels in knee OA patients with advanced-stage (Kellgren and Lawrence, KL grade 3–4) compared with those with early-stage (KL grade 1–2), in addition to marked up-regulation of *CLU* mRNA expression in the inflamed synovial tissues of knee OA patients when compared with the non-inflamed synovium^18^.

Possible associations of CLU mRNA and protein levels with severity of synovitis in knee OA patients were subsequently examined, as illustrated in Table 1. Spearman’s rho correlation analysis uncovered a positive correlation between relative *CLU* mRNA expression and degree of synovitis (*r* = 0.88, *P* < 0.001). Likewise, plasma and synovial fluid CLU levels were both associated with synovitis severity (*r* = 0.56, *P* < 0.001; *r* = 0.75, *P* < 0.001, respectively). After adjusting for age, gender, and BMI, multivariate logistic regression analysis attested a direct link between *CLU* mRNA expression in knee OA synovium and degree of synovitis (β coefficient = 0.126, 95% CI 0.098–0.173, *P* < 0.001). Consistent with this, plasma and synovial fluid CLU levels were shown to be independently correlated with degree of synovitis (β coefficient = 0.004, 95% CI 0.001–0.007, *P* = 0.017; β coefficient = 0.005, 95% CI 0.003–0.006, *P* < 0.001, respectively).

**Table 1:** Spearman's correlation and multivariate linear regression analysis of synovitis severity estimates.

Variables	Degree of synovitis (0–9 points)			
	Spearman's rho correlation		Linear regression^a^	
	Coefficient (*r*)	*P*-value	β coefficient (95% CI)	*P*-value
Age (years)	0.05	0.76	**–**	**–**
Gender (female/male)	− 0.05	0.78	**–**	**–**
BMI (kg/m^2^)	0.18	0.33	**–**	**–**
*CLU* mRNA expression	0.88	**< 0.001**	0.126 (0.098–0.173)	**< 0.001**
Plasma CLU (μg/mL)	0.56	**< 0.001**	0.004 (0.001–0.007)	**0.017**
Synovial fluid CLU (μg/mL)	0.75	**< 0.001**	0.005 (0.003–0.006)	**< 0.001**

*BMI* body mass index, *CLU* clusterin.

Significant values are in bold.

^a^The coefficient was adjusted for age, gender, and BMI.

### CLU alleviated IL-1β-induced cytotoxicity in knee OA FLSs

**Figure 3:**
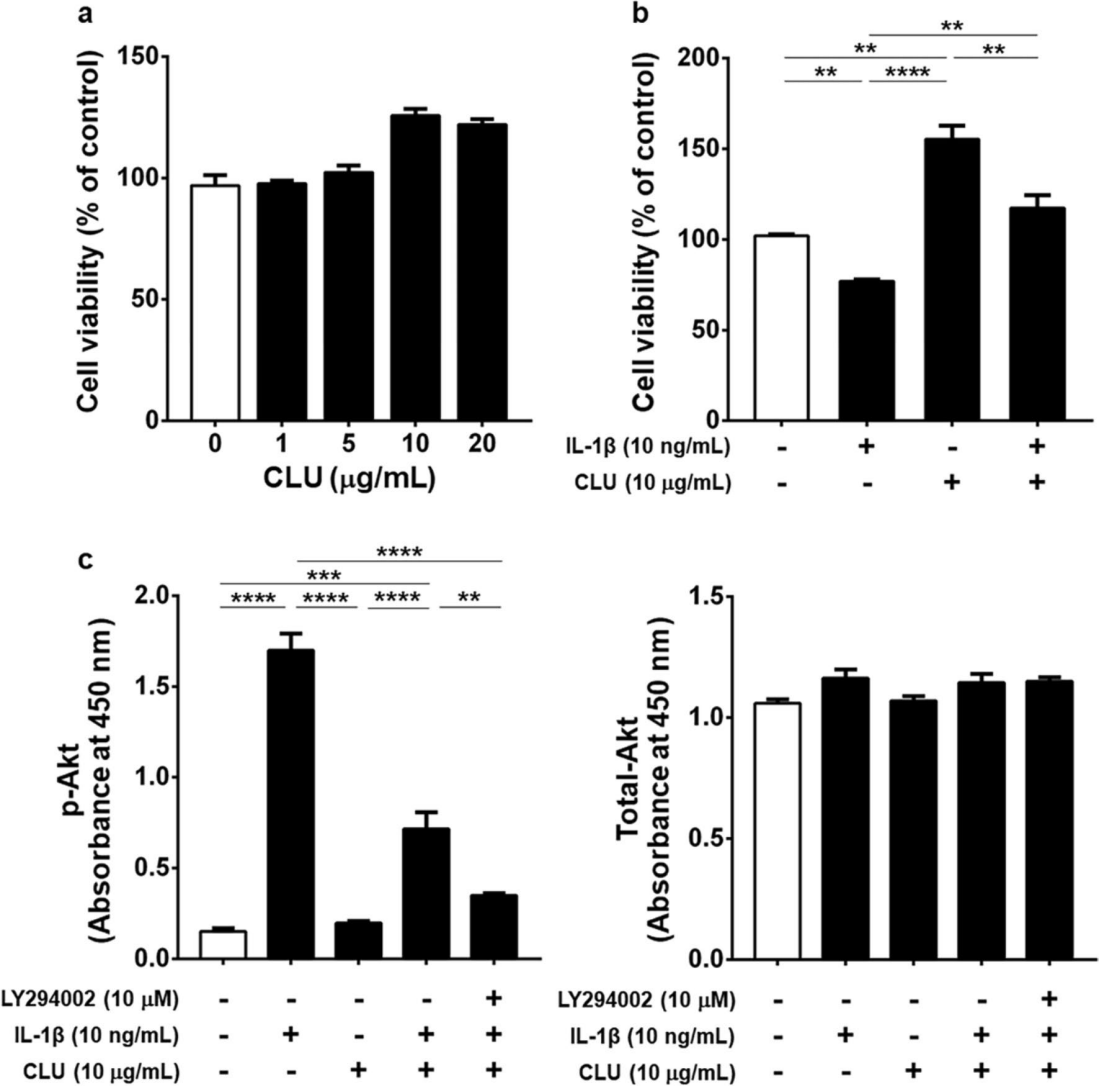
Cell viability and Akt phosphorylation in knee OA FLSs. (**a**) Viability of knee OA FLSs treated with CLU at various concentrations (1, 5, 10, 20 μg/mL). (**b**) Viability of knee OA FLSs treated with either IL-1β in the absence or presence of CLU or CLU alone. (**c**) Phosphorylated and total Akt levels in knee OA FLSs treated with IL-1β in the absence or presence of CLU, CLU alone, or PI3K inhibitor combined with IL-1β and CLU. **P < 0.01, ***P < 0.001, ****P < 0.0001 for comparisons among treatment groups.

Owing to CLU overexpression in knee OA synovitis, an in vitro study was conducted to determine whether CLU had a potential effect against inflammation in knee OA, in which FLSs were isolated from 6 knee OA patients (mean age 71.5 ± 6.9 years, 4 women and 2 man). Primarily, the effect of CLU on cell viability of knee OA FLSs was investigated using MTT assay. As revealed in Fig. 3a, knee OA FLSs stimulated with CLU at various concentrations (1, 5, 10, and 20 µg/mL) showed a tendency for increased cell viability at 24 h when compared to the untreated cells, but these differences were not statistically significant. Particularly, the cells treated with CLU at concentration of 10 μg/mL showed the highest cell viability levels, in which 10 μg/mL CLU as an appropriate concentration was used for our subsequent experiments.

To further determine the effect of CLU on IL-1β-induced cell cytotoxicity, knee OA FLSs were treated with IL-1β, CLU, IL-1β-CLU combination, or without any treatment for 24 h. Compared to the untreated cells, cell viability of knee OA FLSs treated with either CLU or IL-1β-CLU combination was significantly increased (*P* < 0.01, *P* < 0.01, respectively), but remarkably decreased after treatment with IL-1β (*P* < 0.01) (Fig. 3b), suggesting the protective effect of CLU against IL-1β-induced cytotoxicity in knee OA FLSs.

### CLU mitigated IL-1β-induced Akt phosphorylation in knee OA FLSs

Given that IL-1β-induced production of various inflammatory mediators and cartilage-degrading factors reportedly activated Akt through a PI3K-dependent manner, we therefore investigated whether CLU could impair activation of PI3K/Akt pathway induced by IL-1β in knee OA FLSs. Knee OA FLSs were pre-incubated with PI3K inhibitor (LY294002) and thereafter treated with IL-1β in the absence or presence of CLU and CLU alone for 24 h. Compared to the untreated cells, phosphorylated Akt level, an indication of PI3K/Akt activation, was significantly escalated in knee OA FLSs following IL-1β treatment (*P* < 0.0001), but significantly decreased in the presence of either CLU or LY294002 combined with CLU (*P* < 0.001, *P* < 0.0001, respectively). Treatment with CLU alone was observed to effectively prohibit Akt activation, compared with IL-1β-treated group (*P* < 0.0001) (Fig. 3c). These data provided a new understanding that CLU highly impeded IL-1β-induced Akt activation in knee OA FLSs.

### CLU aggravated IL-1β-induced production of ROS and inflammatory cytokine in knee OA FLSs

**Figure 4:**
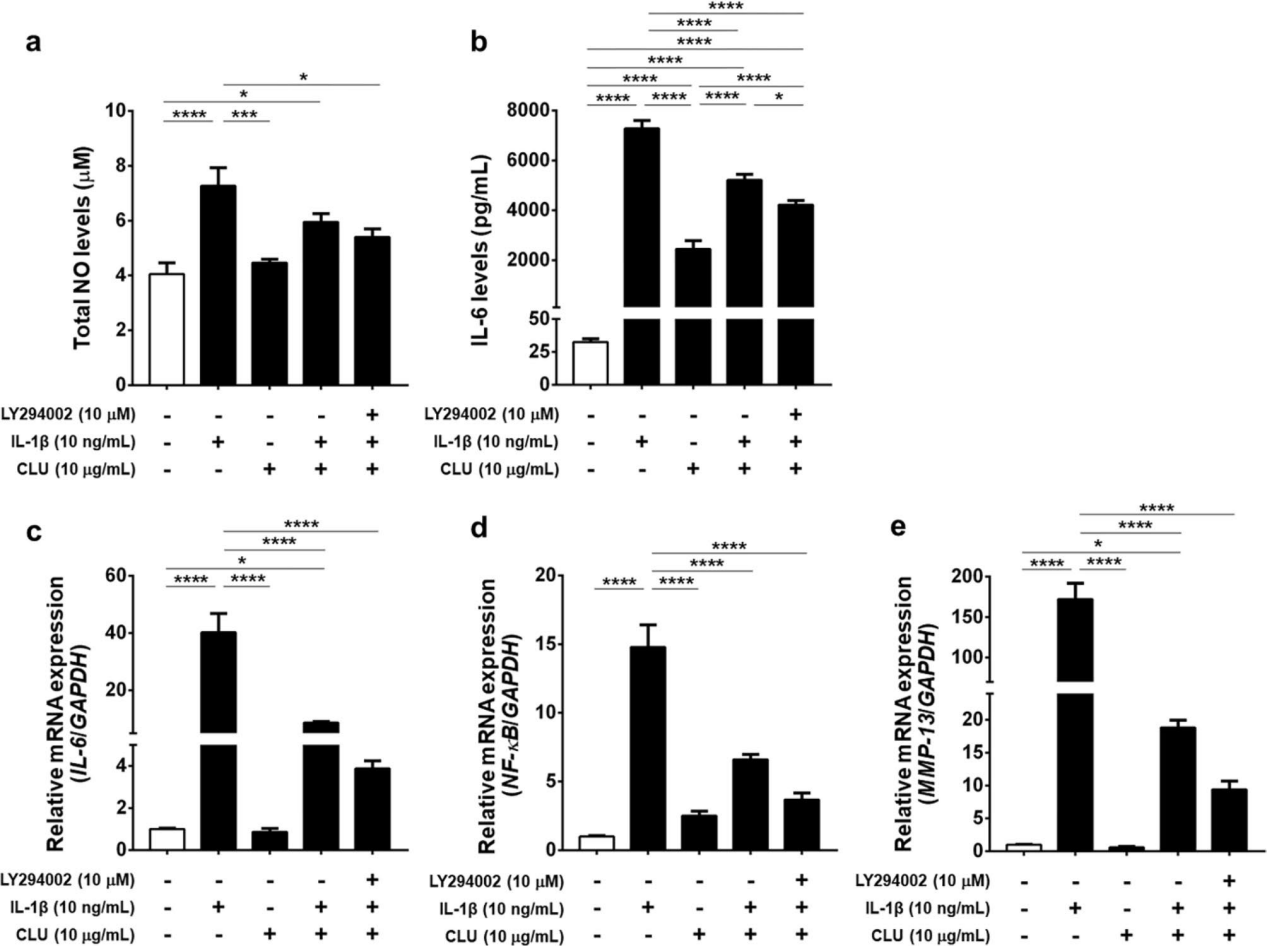
Biochemical and mRNA expression analyses in knee OA FLSs. (**a**) Production of ROS (total NO) in knee OA FLSs stimulated with IL-1β in the absence or presence of CLU, CLU alone, or PI3K inhibitor combined with IL-1β and CLU. (**b**) Production of inflammatory cytokine (IL-6). (**c**) Relative mRNA expressions of *IL-6*. (**d**) *NF-κB*. (**e**) *MMP-13*. *P < 0.05, **P < 0.01, ***P < 0.001, ****P < 0.0001 for comparisons among treatment groups.

Excessive production of ROS and inflammatory cytokine is recognized as a critical pathologic feature driving progression of synovitis in knee OA. We therefore investigated whether CLU quelled production of ROS and inflammatory mediator in knee OA FLSs, in which total NO and IL-6 levels were measured using ELISA. As shown in Fig. 4a, total NO levels were shown to be significantly increased in knee OA FLSs stimulated with IL-1β, compared with those with CLU treatment and the untreated cells (*P* < 0.001, *P* < 0.0001, respectively). Instead, treatment with IL-1β-CLU combination significantly decreased total NO levels in knee OA FLSs, compared to the untreated group (*P* < 0.05). Besides this, knee OA FLSs treated with IL-1β in the presence of LY294002 and CLU had significantly decreased total NO levels, as compared with IL-1β-treated group (*P* < 0.05). In conjunction with the above-mentioned results, compared to knee OA FLSs without any treatment, IL-6 levels were significantly increased in those with IL-1β treatment (*P* < 0.0001), but significantly decreased after stimulation with LY294002 along with IL-1β and CLU (*P* < 0.001), IL-1β-CLU combination (*P* < 0.0001), or CLU alone (*P* < 0.0001) (Fig. 4b). After treatment with a combination of IL-1β and CLU, IL-6 levels were significantly declined in knee OA FLSs when compared to the IL-1β-treated cells (*P* < 0.0001), in accordance with knee OA FLSs treated with either CLU (*P* < 0.0001) or LY294002 together with IL-1β and CLU (*P* < 0.0001) (Fig. 4b). These findings suggest the ability of CLU to prevent IL-1β-induced production of ROS and inflammatory cytokine in knee OA FLSs through inhibiting activation of PI3K/Akt signaling pathway.

### CLU attenuated IL-1β-induced up-regulation of inflammatory and catabolic gene expressions in knee OA FLSs

To evaluate mRNA expressions of inflammatory and catabolic molecules in each treatment group, we additionally performed quantitative real-time PCR. Relative mRNA expressions of inflammatory and catabolic genes are revealed in Fig. 4c–e. The analysis showed that relative mRNA expressions of genes involved in inflammation (*IL-6* and *NF-κB*) and cartilage catabolism (*MMP-13*) were significantly up-regulated in knee OA FLSs following IL-1β treatment, compared to untreated knee OA FLSs (*P* < 0.0001, *P* < 0.0001, *P* < 0.0001, respectively). On the other hand, significant decreases in relative mRNA expressions of *IL-6*, *NF-κB*, and *MMP-13* were observed in knee OA FLSs upon addition of CLU (*P* < 0.0001, *P* < 0.0001, *P* < 0.0001, respectively), IL-1β-CLU combination (*P* < 0.0001, *P* < 0.0001, *P* < 0.0001, respectively), and LY294002 along with IL-1β and CLU (*P* < 0.0001, *P* < 0.0001, *P* < 0.0001, respectively) when compared with IL-1β-treated group. The above-mentioned findings indicate that CLU could constrain up-regulated mRNA expressions of inflammatory mediators and cartilage-degrading factor in knee OA FLSs stimulated with IL-1β via suppressing PI3K/Akt signaling pathway.

## Discussion

Synovitis being important in cartilage degradation offers a target for knee OA treatment, for which molecules known to participate in inflammation appear to have therapeutic value against joint dysfunction and eventual cartilage loss. In the present study, we found that a multifaceted protein related to apoptosis and inflammation^16,19–21^, CLU, was expressed in the systemic and local joint environment of knee OA patients, especially the patients with high-grade synovitis and was positively associated with degree of synovitis. This report uncovered for the first time that CLU efficiently attenuated IL-1β-induced inflammation in knee OA FLSs through inhibiting production of NO and IL-6, in addition to mRNA expressions of *IL-6*, *NF-κB*, and *MMP-13*, which was mediated by regulating PI3K/Akt signaling pathway. Taken together, our data support the notion that CLU could exert its anti-inflammatory action in synovitis and might have potential as a novel therapeutic target for preventing or delaying disease progression of knee OA.

**Figure 5:**
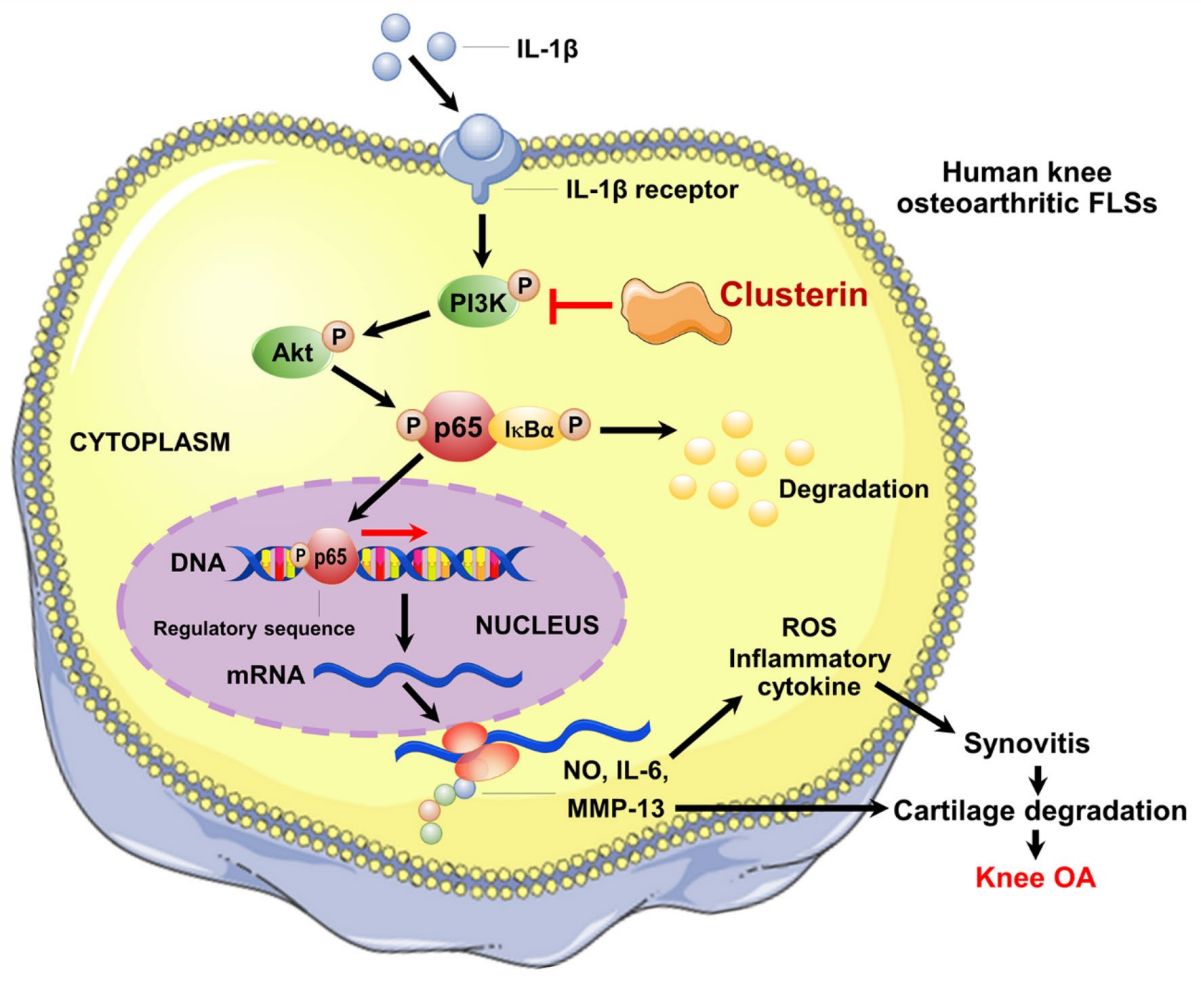
Molecular mechanism underlying anti-inflammatory effect of CLU in knee OA FLSs. CLU has been reported to protect against IL-1β-induced production of ROS, inflammatory cytokine, and cartilage-degrading enzyme in knee OA FLSs through suppressing activation of PI3K/Akt signaling pathway, which may further impede progression of synovitis and subsequent cartilage degradation in knee OA.

As an anti-inflammatory and anti-apoptotic molecule, it is not unexpected that CLU may have an immense potential to be a biologically active mediator for synovitis-induced joint degeneration in arthritic patients. Supporting this, a number of clinical studies unveiled significant elevations in CLU levels in the circulation and synovial fluid of OA^18,22–24^, in addition to up-regulation of its mRNA expression in the cartilage and the synovium of arthritic patients^16,18,22,25^. These previous results were confirmed by the present study, which denoted up-regulation of CLU protein and mRNA expressions in knee OA synovium, particularly in the inflamed synovium and remarkable increases in its protein levels in plasma and joint fluid of knee OA patients with synovitis. More specifically, it has been shown that CLU mRNA and protein levels were both associated with synovitis severity in knee OA. All our findings led us to postulate that increases in CLU mRNA and protein levels in the systemic and local joint environment would reflect severity synovitis of knee OA. This assumption was addressed by our subsequent analysis, showing positive links between *CLU* mRNA expression and mRNA expressions of signaling molecules implicated in inflammatory response including *IL-6*, *NF-κB*, and *MMP-13* in the synovium of knee OA patients. However, whether these associations reflected a causal role for synovitis in knee OA remains unclear. To address this challenge, we conducted an in vitro study to elucidate the protective effect of CLU against inflammation in knee OA FLSs and also found that CLU inhibited IL-1β-induced cell cytotoxicity. Besides its beneficial effect on viability of knee OA FLSs, CLU treatment has been shown to prohibit IL-1β-induced production of NO and IL-6 and impede up-regulation of *IL-6*, *NF-κB*, and *MMP-13* mRNA expressions in knee OA FLSs pre-treated with IL-1β. In accordance with our aforementioned findings, a previous study by Falgarone et al.^26^. showed significant inflammatory action of CLU in rheumatoid synovitis, by which silencing expression of *CLU* using small interfering RNA altered expressions of numerous genes related to progression of synovitis in RA FLSs in response to tumor necrosis factor (TNF)-α stimulation. Similarly, a previous study by Devauchelle et al.^16^. displayed that *CLU* knock-down by siRNA transfection significantly enhanced release of inflammatory cytokines including IL-6 and IL-8 in RA FLSs. Collectively, our findings derived from an in vitro study shed light on the protective effect of CLU against IL-1β-induced inflammation in knee OA FLSs via suppressing production of inflammatory and matrix-degrading molecules at mRNA and protein levels, which may help explain overexpression of CLU mRNA and protein associated with synovitis severity in knee OA patients. However, the exact signaling mechanisms governing CLU anti-inflammatory function in knee OA synovitis become far less clear. In the light of current knowledge, CLU has been clearly evinced to inhibit inflammatory response via NF-κB signaling mediated through modulating IκB stability^15,16,20,21^. It has been well-recognized that NF-κB, a key player in the distinctive inflammatory processes, plays a critical role in knee OA pathogenesis^27^, through which NF-κB activation influenced by IL-1β causes inflammation and cartilage destruction by release of chemokines, cytokines, and matrix-degrading enzymes such as NO, IL-6, and MMPs^19,28^. Indeed, a recent experimental study demonstrated that CLU secretion was regulated by pro-inflammatory cytokine including IL-1β in equine models of cartilage degradation^29^. Since PI3K/Akt is an upstream molecule of NF-κB pathway, blocking phosphorylation of Akt can inhibit NF-κB activation and subsequently eliminate production of inflammatory mediators and matrix-degrading molecules^11,30^. It has been discovered that CLU exerted its anti-apoptotic action through PI3K/Akt signaling pathway^31^. The aforementioned findings make PI3K/Akt an attractive cascade for CLU anti-inflammatory action in knee OA FLSs and for attenuating knee OA development. Supporting this, our additional results showed that both CLU and PI3K inhibitor clearly mitigated IL-1β-induced Akt phosphorylation in knee OA FLSs, which further quelled transcriptional expressions of inflammatory mediators (*IL-6* and *NF-κB*) and catabolic molecule (*MMP-13*) and eventually hindered translational production of inflammatory molecules (NO and IL-6). Altogether, these results provided novel insights into the inhibitory effect of CLU against IL-1β-induced production of inflammatory mediators and cartilage-degrading factors at mRNA and protein levels in knee OA FLSs via constraining activation of PI3K/Akt signaling pathway. The molecular mechanism underlying anti-inflammatory function of CLU in knee OA FLSs is delineated in Fig. 5.

Albeit significant findings derived from clinical and in vitro studies, this study inevitably had some inherent limitations that need to be taken into account. The most important caveat is the fact that the effect of CLU on inflammation in normal human FLSs was unachievable, which may obviate determination on its protective effect against inflammation under normal condition. Furthermore, the role of other inflammatory cytokines such as TNF-α in prompting inflammation in knee OA FLSs remains to be determined. Additionally, as PI3K inhibitor was unable to completely prevent against IL-1β-induced overexpression of inflammatory mediators when compared to untreated knee OA FLSs, it is important to note that additional molecular mechanisms behind CLU role in knee OA FLSs still need to be elucidated.

## Conclusions

This study provided supporting evidence for our previous study^18^ that CLU mRNA and protein were highly expressed in the systemic and local joint environment of knee OA patients with synovitis and were both associated with degree of synovitis. Apart from this, the present in vitro study supports the significant involvement of CLU in inflammatory response, in which CLU has been shown to subdue production of NO and IL-6 induced by IL-1β in knee OA FLSs, in addition to mRNA expressions of *IL-6*, *NF-κB*, and *MMP-13*. All these findings from both clinical and in vitro studies indicate the ability of CLU to constrain IL-1β-induced inflammation in human knee OA FLSs via regulating PI3K/Akt pathway. Further studies are warranted to better understand alternative mechanisms of CLU anti-inflammation role in knee OA synovitis, which would open up the opportunity for novel therapeutic targets aimed at counteracting synovitis and eventual cartilage destruction in knee OA.

### Supplementary Information

Supplementary Tables.

## Data Availability

The data that support the findings of this study are available on reasonable request from the corresponding authors.

## Abbreviations

ACR: American College of Rheumatology
ANOVA: One-way analysis of variance
BMI: Body mass index
CLU: Clusterin
DMEM: Dulbecco’s modified Eagle medium
ELISA: Enzyme-linked immunosorbent assay
FBS: Fetal bovine serum
FLSs: Fibroblast-like synoviocytes
GADPH: Glyceraldehyde 3-phosphate dehydrogenase
H&E: Hematoxylin and eosin
IL: Interleukin
MMP: Matrix metalloproteinase
MTT: 3-(4,-Dimethylthiazol-2-y)-2,5-diphenyl-tetrazolium bromide
NF-κB: Nuclear factor-kappa B
NO: Nitric oxide
OA: Osteoarthritis
PBS: Phosphate buffer solution
PCR: Polymerase chain reaction
PI3K: Phosphatidylinositol-3-kinase
RA: Rheumatoid arthritis
ROS: Reactive oxygen species
SD: Standard deviation
SPSS: Statistical package for social sciences
TKR: Total knee replacement
TNF: Tumor necrosis factor
