# Author Correction: Clusterin exacerbates interleukin-1β-induced inflammation via suppressing PI3K/Akt pathway in human fibroblast-like synoviocytes of knee osteoarthritis

**DOI:** 10.1038/s41598-024-63664-x

**Published:** 2024-06-05

**Authors:** Tachatra Ungsudechachai, Sittisak Honsawek, Jiraphun Jittikoon, Wanvisa Udomsinprasert

**Affiliations:** 1 Department of Biochemistry, Faculty of Pharmacy, Mahidol University, 447 Sri-Ayudthaya Road, Rajathevi, Bangkok, 10400 Thailand; 2 Department of Biochemistry, Osteoarthritis and Musculoskeleton Research Unit, Faculty of Medicine, Chulalongkorn University, King Chulalongkorn Memorial Hospital, Thai Red Cross Society, Bangkok, Thailand

Correction to: *Scientific Reports* 10.1038/s41598-022-14295-7, published online 15 June 2022

The original version of this Article contained an error. In the Methods section, under the subheading ‘Patients and Synovial Biopsies’, the approval number of the Institutional Review Board from Mahidol University was incorrect.

“The study protocol conducted in conformity with the guidelines of the Declaration of Helsinki was approved by the Institutional Review Board of the Faculty of Medicine, Chulalongkorn University (IRB number 533/54) and the Faculty of Dentistry/Faculty of Pharmacy, Mahidol University (IRB number 2018/072.1812). All participants were fully informed regarding the study protocol and procedures prior to entering the study. Written informed consent was obtained from the participants.”

now reads:

“The study protocol conducted in conformity with the guidelines of the Declaration of Helsinki was approved by the Institutional Review Board of the Faculty of Medicine, Chulalongkorn University (IRB number 533/54) and the Faculty of Dentistry/Faculty of Pharmacy, Mahidol University (MU-DT/PY-IRB 2019/074.2511). All participants were fully informed regarding the study protocol and procedures prior to entering the study. Written informed consent was obtained from the participants.”

The original Article has been corrected.

