# Supplementary material for: Clusterin exacerbates interleukin-1β-induced inflammation via suppressing PI3K/Akt pathway in human fibroblast-like synoviocytes of knee osteoarthritis
Source: Sci Rep. 2022 Jun 15;12:9963. doi: 10.1038/s41598-022-14295-7 (PMC9200742; doi:10.1038/s41598-022-14295-7)
Supplement: Supplementary file 1 — Supplementary Tables. [file 41598_2022_14295_MOESM1_ESM.docx]

**Supplementary table 1** Primers used for mRNA expression analyses.

| **Genes** | **Primers** | **Sequence** |
| --- | --- | --- |
| ***CLU*** | Forward | 5’-GCGAAGACCAGTACTATCTG-3’ |
|  | Reverse | 5’-TTTTGCGGTATTCCTGCAGC -3’ |
| ***IL-6*** | Forward | 5′-AGCCACTCACCTCTTCAGAAC-3′ |
|  | Reverse | 5′-ACATGTCTCCTTTCTCAGGGC-3′ |
| ***NF-κB*** | Forward | 5′-GACCGCTGCATCCACAGTTT-3′ |
|  | Reverse | 5′-GGATGCGCTGACTGATAGCC-3′ |
| ***MMP-13*** | Forward | 5′-TTGCAGAGCGCTACCTGAGATCAT-3′ |
|  | Reverse | 5′-TTTGCCAGTCACCTCTAAGCCGAA-3′ |
| ***GAPDH*** | Forward | 5’-GTGAAGGTCGGAGTCAACGG-3’ |
|  | Reverse | 5’-TCAATGAAGGGGTCATTGATGG-3’ |

Abbreviations: CLU, clusterin; GAPDH, glyceraldehyde 3-phosphate dehydrogenase; IL-6, interleukin-6; MMP-13; matrix metallopeptidase-13; NF-κB; nuclear factor kappa B.

**Supplementary table 2** Baseline characteristics of knee OA patients.

| **Variables** | **Knee OA patients** | | | | ***P*-value** |
| --- | --- | --- | --- | --- | --- |
|  | **Total** | **No synovitis** | **Low-grade synovitis** | **High-grade synovitis** |  |
| Number | 50 | 16 | 19 | 15 |  |
| Age (years) | 72.2±8.0 | 74.0±6.3 | 70.8±8.6 | 73.3±8.2 | 0.57 |
| Gender (female/male) | 38/12 | 12/5 | 14/4 | 12/3 | 0.84 |
| BMI (kg/m^2^) | 24.9±3.5 | 24.0±3.4 | 25.0±2.8 | 25.9±4.7 | 0.53 |

Abbreviations: BMI, body mass index; OA, osteoarthritis.
